# Success Rate of Split-Thickness Skin Grafting of Chronic Venous Leg Ulcers Depends on the Presence of *Pseudomonas aeruginosa*: A Retrospective Study

**DOI:** 10.1371/journal.pone.0020492

**Published:** 2011-05-31

**Authors:** Trine Høgsberg, Thomas Bjarnsholt, Jens Schiersing Thomsen, Klaus Kirketerp-Møller

**Affiliations:** 1 Copenhagen Wound Healing Centre, Bispebjerg Hospital, Copenhagen, Denmark; 2 Institute for International Health, Immunology and Microbiology, University of Copenhagen, Copenhagen, Denmark; 3 Department of Clinical Microbiology, Rigshospitalet, Copenhagen, Denmark; 4 Orthopedic Department, Hvidovre University Hospital, Copenhagen, Denmark; Charité, Campus Benjamin Franklin, Germany

## Abstract

The last years of research have proposed that bacteria might be involved in and contribute to the lack of healing of chronic wounds. Especially it seems that *Pseudomonas aeruginosa* play a crucial role in the healing. At Copenhagen Wound Healing Centre it was for many years clinical suspected that once chronic venous leg ulcers were colonized (weeks or months preoperatively) by *P. aeruginosa*, the success rate of skin grafting deteriorated despite aggressive treatment. To investigate this, a retrospective study was performed on the clinical outcome of 82 consecutive patients with chronic venous leg ulcers on 91 extremities, from the 1^st^ of March 2005 until the 31^st^ of August 2006. This was achieved by analysing the microbiology, demographic data, smoking and drinking habits, diabetes, renal impairment, co-morbidities, approximated size and age of the wounds, immunosuppressive treatment and complicating factors on the clinical outcome of each patient. The results were evaluated using a Student T-test for continuous parameters, chi-square test for categorical parameters and a logistic regression analysis to predict healing after 12 weeks. The analysis revealed that only 33,3% of ulcers with *P. aeruginosa*, isolated at least once from 12 weeks prior, to or during surgery, were healed (98% or more) by week 12 follow-up, while 73,1% of ulcers without *P. aeruginosa* were so by the same time (p = 0,001). Smoking also significantly suppressed the outcome at the 12-week follow-up. Subsequently, a logistic regression analysis was carried out leaving *P. aeruginosa* as the only predictor left in the model (p = 0,001). This study supports our hypothesis that *P. aeruginosa* in chronic venous leg ulcers, despite treatment, has considerable impact on partial take or rejection of split-thickness skin grafts.

## Introduction

In Denmark, it has been estimated that the prevalence of non-healing wounds is about 1% of the population. The prevalence and incidence of leg ulcers are similar to elsewhere in the industrialized world[1]. This has large socio-economic costs. The total expenses for treatment of wounds are estimated to be approximately 2% to 3% of the total budget of the health care system in Denmark[2]. Meshed Split Thickness Skin Grafts (STSGs) are widely used in the treatment of non-healing leg ulcers. The technique of meshing STSGs was first reported in 1964[3]. The clinical outcome of skin grafting depends on a variety of factors, some more substantiated than others. Only a limited number of publications on skin graft loss due to infection exist and they are mostly related to the management of burn wounds. A number of the studies have focused on the importance of quantitative rather than qualitative bacteriology others vice versa. From the literature it can be deducted that a successful skin graft “take” is less likely to occur on experimental or clinical wounds that contain more than 10^5^ viable bacteria per gram of tissue[4], [5]. The majority of publications discussing qualitative bacteriology point out haemolytic streptococci in particular *Streptococcus pyogenes* as the predominant species leading to graft lysis[6]–[10]. Graft lysis due to non-group A beta-haemolytic streptococci has also been reported[11] together with *Staphylococcus aureus* [12] and *Pseudomonas aeruginosa* [8], [12]–[16].

Skin graft failure due to P. aeruginosa is not a novel hypothesis. It was proposed in 1951[17]. Since then, there have been only few articles published concerning this issue. The hypothesis was confirmed by Gilliland et al[12] more than 20 years ago, stating the isolation of Pseudomonas from an ulcer prior to skin grafting significantly impairs skin graft take.

Despite this knowledge it still seems to be a problem of high relevance.

**Figure 1:**
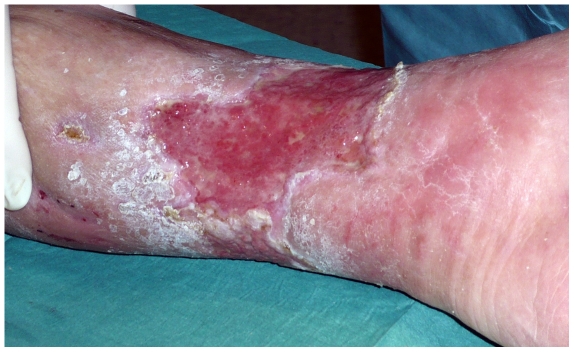
A venous leg ulcer at time of operation. Standard state-of-the-art treatment failed to heal the ulcer and according to our protocol the patient was offered a revision and split-skin transplant.

It is our clinical experience during our daily work with chronic venous leg ulcers and STSGs that once the ulcers are colonized by *P. aeruginosa*, even weeks or months preoperatively, the success rate of skin grafting deteriorates. This, in spite of an aggressive treatment, in our clinic, of *P. aeruginosa,* in the form of extensive debridement of the wound bed before grafting often combined with systemic antibiotic therapy. Our focus on *P. aeruginosa* has increased over time due to increasing evidence of this bacterium to impair healing of chronic wounds. From our recent studies we have elucidated *P. aeruginosa* as being present in the wounds as biofilms in many of our patients, see Figure 1 [18]–[20]. The ability of colonizing bacteria to establish themselves and proliferate in a biofilm could be the cause of the lack of successful antibiotic treatment. Currently we are changing our antibiotic regime to accommodate the eradication of bacteria in biofilms. In order to evaluate the impact of *P. aeruginosa* on graft take in our present antibiotic regimen we chose to make a retrospective study to establish the relevance of the subject prior to further investigation. Our aim of this study was to determine whether graft take and survival of the graft, in the first 3 months, was dependent on wound bed bacteriology from 12 weeks prior, to or during surgery, with particular attention to *P. aeruginosa.*

## Materials and Methods

### Patients

A retrospective study was conducted of consecutive patients who underwent meshed split-thickness skin grafting for chronic venous leg ulcers in Copenhagen Wound Healing Centre, Bispebjerg Hospital, Denmark, over a period of 18 months from the 1^st^ of March 2005 until the 31^st^ of August 2006. The centre consists of a team of expert wound care specialists[1], [9]. Data was collected from the medical records.

Ninety-five patients received autografting with meshed split-skin transplants of their lower leg ulcers of venous origin as the apparent aetiology. Thirteen of the 95 patients were excluded in our analysis. The main reason being lack of data due to their absence at the 12-week follow-up: Six patients passed away for unrelated causes in the weeks following surgery. One patient did not attend the outpatient clinic until after 9 months and 3 patients only attended it once or twice but missed out on the final appointments including the 12-week follow-up. Moreover, 2 patients exhibited pronounced non-compliance, which led to immediate rejection of the transplants and, finally, one patient was excluded due to a total lack of microbiology data. A total of 9 of the 82 patients included in our analysis required a second or third grafting during the study period for the treatment of recurrent or new ulcers. Only the first ulcer episode was included. Of the included patients, 22 had bilateral ulcers. Of these, only 9 patients were included in the study with bilateral ulcers and the remaining 13 were included with unilateral ulcers due to insufficient data as regards to which leg the microbiology was achieved from. This selection enabled us to perform an exact statement on whether or not *P. aeruginosa* had been isolated from that particular leg. Cases of multiple ulcers and concurrent grafts on one leg were treated as a single event. In about 70% of the cases there was only 1 ulcer per leg. As to the other listed bacteria in the bilateral cases, it has been necessary to assume, that the microbiology assessed was identical in the 2 extremities. Overall the study includes 82 patients (91 extremities).

Venous origin of the ulcers was classified according to a history of past thrombophlebitis or varicectomy and characteristic clinical signs of venous insufficiency. Moreover, the origin was often confirmed by Colour Duplex Vein scan findings[21]. In case of absent pedal pulse, we defined sufficient blood supply (by stain gauge) as an ankle pressure more than 70 mmHg and/or toe pressure more than 50 mmHg, taking the location of the wounds into account.

### Ethics

An application was sent to the Ethical Comity for Copenhagen and the Danish National Board of Health and the present study did not need approval as only anonymous data were used and that no informed consents from the patients were needed.

### Grafting

**Figure 2:**
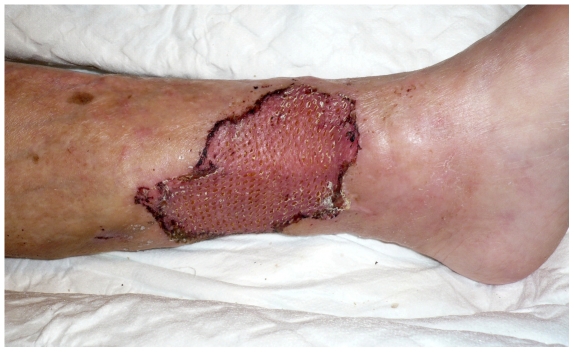
The same ulcer as **Figure 1** five weeks post operative. The spit-skin transplant is well attached and almost completely healed.

Our grafting methods and procedures have been described previously by Bitsch et al[9] but are briefly as follows: tangential excision of their leg ulcers followed by autografting with meshed STSGs (Figure 1 and 2). This entailed mechanical debridement of necrotic tissues, lipodermatosclerotic skin, exposed tendons, subcutaneous calcifications and bone immediately followed by mesh skin grafting. Mesh grafting consists of harvesting a STSG (0,3 mm thick) with a dermatome and expanding this graft with a mesher. Insufficient perforator veins are ligated superficially to the fascia. Donor grafts are obtained from the thigh. In addition, immobilizing devices are used if skin grafts are applied on to mobile surfaces. The vast majority of the patients are discharged after an average period of 7–10 days. Compression therapy is continued after discharge. Early standard postgraft follow-up consists of 3 controls, in the 3^rd^, 6^th^ and 12^th^ week in the outpatient clinic.

### Bacteriology

We have examined the bacteriology assessed from the wounds from 12 weeks prior, to and during surgery. The ulcer surfaces were swabbed with a sterile charcoal swab preoperatively. During surgery, but before grafting and administration of antibiotics, the microbiology assessment was either detected by a charcoal swab or by a biopsy. The swabs or mechanically crushed tissue were cultured on aerobic and anaerobic standard agar plates (Statens Serum Institute [SSI], Copenhagen, Denmark). The aerobic cultures were grown on selective gram-negative plates (blue plates; SSI) as well as 5% horse blood agar (SSI) and incubated at 35°C. The anaerobic cultures were grown on anaerobic plates (SSI) and incubated under anaerobic conditions in anaerobic chamber at 35°C. All plates were inspected on days 1 and 2. Identification of bacteria was performed using standard methods including selective growth on relevant agar plates, VITEK 2 and API diagnostic strips. The following bacteria have been the issue of interest: *P. aeruginosa*, Pseudomonas species, *S. aureus*, haemolytic Streptococci (group A, B, C, G), *Proteus,* gram-negative bacilli and anaerobic bacteria.

### Antibiotic treatment

Systemic antibiotics were provided if necessary and in accordance with the microbiology assessed from the wounds. Routinely either cefuroxime 1500 mg alone or in combination with gentamicin 160–240 mg were administered intravenously as single shots perioperatively. The majority of patients with *P. aeruginosa* either received ciprofloxacin (typically 500 mg orally x 2) alone or in combination with one of the following: meropenem (2 g x 3), a combination of piperacillin and tazobactam (4 g x 3), ceftazidim (1 g x 2) or gentamicin (160–240 mg x 1) intravenously of varying periods. In addition, the majority was treated with silver containing dressings preoperatively.

### Evaluation

The clinical outcome was evaluated, as percentages of graft take observed at the routine check-ups in the first 3 months with emphasis on the 12^th^ week follow-up. Graft take was divided into two groups: *healed* versus *not-healed*, with five subgroups in total. The *healed* category was divided into two subgroups. Completely healed, [100%], and 98% or more, [98%–100%], of the grafted skin intact with defects thought to be insignificant. The *not-healed* category was divided into three subgroups: less than 98% but more than 75% take, [75%–98%], 75% or less but more than 5% take, [5%–75%], and finally 5% or less, [0–5%]. The coding classification for healing is subjective nevertheless it is problematic to use the term *100% healed* when working with STSGs. Technically, it is almost impossible to avoid very small and clinical insignificant defects during the procedure itself and the following rehabilitation. We have chosen 98% or more as a *healed category* with defects thought to be insignificant because that is what seemed reasonable for what could be classified very successful.

Three of the authors (TH, JST and KKM) evaluated the percentages of graft take in order to test the reproducibility of data. The evaluators randomly selected 25% of the cases and achieved conformity of 100%.

The long-term outcome was recorded as the last known condition of the ulcer documented in the medical records until an eventual new operation.

Co-morbidities were identified. The major groups were: ischemic heart disease, chronic obstructive pulmonary disease, stroke with sequelae, congestive heart failure, cancer, rheumatoid arthritis, scleroderma, chronic anaemia and drug addiction. Diabetes mellitus and chronic renal impairment were listed separately together with the use of immunosuppressive drugs (prednisone, ciclosporin or azathioprin).

Sex, age, smoking and drinking habits, approximated age and size of the wounds and complicating factors for wound healing like exposed bone, bare tendon or involvement of the achilles region were registered. Smoking refers to smoking habits on the day of hospitalization, typically a few days prior to surgery. Drinking habits were only recorded if the number of drinks per week was higher than what the Danish Health Department recommends. This is no more than 21 drinks per week for men (max 252 g/wk) and no more than 14 drinks per week for women (max 168 g/wk).

### PNA FISH

**Figure 3:**
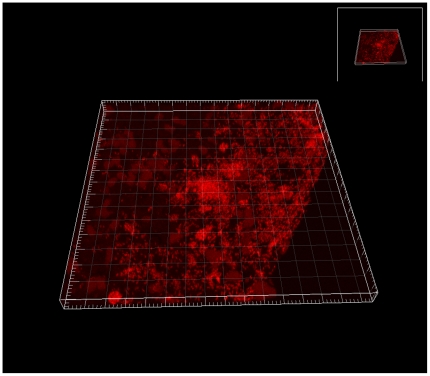
CLSM three-dimensional image of a large *P. aeruginosa* biofilm in the wound bed of a chronic wound (identified by a specific PNA FISH probe (red)).

Tissue section of a chronic wound was analyzed by fluorescence in situ hybridization (FISH) using peptide nucleic acid (PNA) probes (Figure 3). A Texas Red-labeled *P. aeruginosa*-specific PNA probe in hybridization solution (AdvanDx, Inc., Woburn, MA), was added to each section and hybridized in a PNA FISH workstation covered by a lid at 55°C for 90 min. The slides were washed for 30 min at 55°C in wash solution (AdvanDx). Three-dimensional imaging of the bacteria in the wounds was done by confocal laser scanning microscopy (CLSM) using a Zeiss LSM 510 system (Carl Zeiss GmbH, Jena, Germany) equipped with ahelium-neon laser for excitation of the fluorophores. Simulated fluorescence projections of the biofilms were generated by using the IMARIS software package (Bitplane AG). Images were further processed for display by using Photoshop software (Adobe).

### Statistical analysis

The statistical program used was SPSS version 15.0. Patient and wound characteristics were compared between the groups by means of Student T-test for continuous parameters and chi-square (or Fisher Exact) test for categorical parameters. Finally, a logistic regression analysis was performed using forward stepwise method to predict healing after 12 weeks.

## Results

The patients studied consist of 82 patients (91 extremities) with 45 women (54,9%) and 37 men (45,1%). The average (SD) age was 69,5 (13,5) years ranging from 39 to 92 years. Diabetes was present in 15 patients (18,3%). Twenty patients were smokers (24,7%) and 10 patients (12,2%) had excessive alcohol consumption as described earlier. The average interval between grafting and the 12-week follow-up was 13,0 (2,8) weeks ranging from 7–22 weeks. The average interval from grafting to “long-term outcome” was 57,4 (33,5) weeks ranging from 11–145 weeks. The approximated average age of the ulcers before surgery was 12,8 (15,7) months (range: 3 weeks–9 years) and the approximated average size of a single (if solitary) or concomitant ulcers on one extremity was 102,6 (118,1) cm2 (range: 3 cm2–600 cm2).

### Healing and bacteriology

As for the healing, interestingly only 33,3% (8/24) of ulcers with *P. aeruginosa* (isolated at least once in the 12 weeks prior, to or during surgery) were healed (98% or more) by week 12 follow-up, while 73,1% (49/67) of ulcers without *P. aeruginosa* were so by the same time (p = 0,001, Chi-square test), see table 1. Looking at the more detailed healing categories we also found significant differences (p = 0,011, Mann-Whitney), see table 2. We have used a categorical ordinal scale to increase sensitivity in the analyses done (compared to the merged categories healed/not healed). The analysis of the detailed data shown in table 2 point in the same direction as the analysis of the merged categories in table 1 as pointed out in the article.

**Table 1:** Healing data of ulcers 12 weeks postoperative from ulcers with or without *P. aeruginosa.*

Week 12 follow-up results:	*P. aeruginosa* pre- or perioperative	
	No	Yes
Healed	73,10%	33,30%
[98%-100%]	(49/67)	(8/24)
Not healed	26,90%	66,70%
[0%-98%]	(18/67)	(16/24)

Table 2 is transferred into table 1 by merging categories.

P = 0,001, N = 91, Chi = 11,96, Chi-square test.

**Table 2:** Detailed healing categories at 12 weeks follow-up divided by presence of *Pseudomonas aeruginosa* pre- and/or peri-operative or not.

	*P. aeruginosa* pre- and/or perioperative	
Week 12 follow-up results:	N = 67	N = 24
	No	Yes
[0%-5%] healed	10,40%	33,30%
[ 5%-75%] healed	7,50%	12,50%
[75%-98%] healed	9,00%	20,80%
[98%-100%] healed	23,90%	0,00%
[100%] healed	49,30%	33,30%

P = 0,011, Mann Whitney test.

No statistically significant correlation could be detected between the clinical outcome and the other microorganisms listed; *S. aureus,* Pseudomonas species, anaerobic bacteria, *haemolytic Streptococci, Proteus* and gram-negative bacilli. Neither did age, sex, alcohol consumption, diabetes, renal impairment, co-morbidities, approximated size and age of the wounds, immunosuppressive treatment or complicating factors. Moreover, these entities were evenly distributed in the 2 groups (*P. aeruginosa* versus the non-*P. aeruginosa* group) except from smoking and approximated age of wound. On average, ulcers in the *P. aeruginosa* group were 18,3 (18,6) months old versus 10,8 (14,1) months in the non- *P. aeruginosa* group (p = 0,043, Student T-test). Only 20,9% (14/67) of the ulcers found on non-smokers, contained *P. aeruginosa* while 45,5% (10/22) found on smokers (45,5%), had *P. aeruginosa* (p = 0,024, Chi-square test). Smoking was the only entity, that was also significant in regards to the outcome at the 12 week follow-up with 40,9% (9/22) of ulcers on smokers healed versus 68,7% (46/67) of ulcers on non-smokers (p = 0,020, Chi-square test). A logistic regression analysis to predict healing at week 12 was carried out including *P. aeruginosa*, diabetes, alcohol consumption, smoking and sex as predictors. By using the forward stepwise methods on *P. aeruginosa* (p = 0,001, Logistics regression) it was the only predictor left in the model (smoking was not as significant, indicating some correlation between smoking and *P. aeruginosa*).

The perioperative isolation of *P. aeruginosa*, revealed results even more pronounced with only 26,7% (4/15) of ulcers with *P. aeruginosa* healed versus 68,2% (45/66) of them without *P. aeruginosa* healed (p = 0,007, Fisher's Exact test). If *P. aeruginosa* was isolated at least once in the 12 weeks prior to surgery, excluding the perioperative day, similar results occurred, but yet a bit less conspicuous: 38,1% (8/21) healed in the *P. aeruginosa* group versus 67,2% (43/64) in non-*P. aeruginosa* group (p = 0,018, Chi-square test).

The 12 weeks healing corresponded very well to the long-term healing. 82,2% (74/90) of the extremities that were recorded as healed at both time points (N = 47) or not healed at both time points (N = 27) remained so in these categories. The long-term outcome in regards to the *P. aeruginosa* group exclusively showed that 37% of ulcers healed with *P. aeruginosa* versus 68,2% without it (p = 0,009, Chi-square test).

## Discussion

No unifying hypothesis can explain the loss of skin transplants. A wide range of things are believed to adversely influence skin graft take; haematoma or shearing movements[8], inadequate compliance, deficient blood supply, presence of microtrombi in the dermal blood vessels[22], local fibrin deficiency in the wound bed[23] and former thrombophlebitis in relation to primary deep vein incompetence[24] are examples. Skin graft loss due to infection make up only a minor part in the literature with very few publications on deterioration of skin grafts due to *P. aeruginosa* [8], [12]–[16], particularly in the field of chronic lower limb ulcerations. These papers do not reach an agreement on the severity of the role of *P. aeruginosa*. They all concentrate on microbiology assessed immediately prior to grafting or postgrafting. To our knowledge, the only paper with regards to leg ulcers is more than 20 years old[12]. In this paper Gilliland et al reported that the initial swab results (at admission) were not related to the outcome of skin grafting, it was only the presence of bacteria (Pseudomonas and *S. aureus*) in the immediate preoperative or postoperative periods that played a role. Surgical debridement was only executed if necessary, but to what extent and details concerning antibiotics were undocumented. Likewise, Pseudomonas was not subdivided into species and the group of patients was heterogeneous by taking all leg ulcers into account despite aetiology. Unal et al[16] found that *P. aeruginosa* was an equally prominent danger as *S. pyogenes* in skin graft survival in routine plastic surgery practice. They only focused on bacteriological cultures assessed postoperatively and only obtained samples from the defected grafts, thereby fail to elucidate what came first; *P. aeruginosa* or the defects? Mc Gregor,[8] in contrast, claims that infection with *P. aeruginosa* reduces graft take but not to an extent comparable with *S. pyogenes*. He also stated that its presence is a nuisance rather than a disaster and it may reduce graft takes by 5–10% at most.

In our study, the presence of *P. aeruginosa* was by far the best predictive marker for the outcome of STSGs of chronic venous leg ulcers. In other words, if *P. aeruginosa* was isolated at least once in 12 weeks prior, to or during surgery, it was a significant negative predictor for the outcome of the graft. Overall, our results show, that 33,3% of ulcers with *P. aeruginosa* were healed by the 12-week follow-up, while 73,1% of ulcers without *P. aeruginosa* were so by the same time. None of the other bacteria listed showed any significant difference in the clinical outcome. Neither did age, sex, alcohol consumption, diabetes, renal impairment, co-morbidities, approximated size and age of the wounds, immunosuppressive treatment or complicating factors. Smoking was the only entity that was also significant in regards to the outcome at the 12-week follow-up. In this perspective, it is important to remember that almost 50% of the ulcers among smokers had *P. aeruginosa*. A logistic regression analysis to predict healing at week 12 showed *P. aeruginosa* as the only significant predictor left in the model (p = 0,001, Forward stepwise logistic regression), whereas sex, diabetes, alcohol, age and smoking where insignificant. It is generally accepted that smoking impairs wound healing[25]. Furthermore, in our study, ulcers with *P. aeruginosa* were statistically significant older than ulcers from where it had not been isolated. Perhaps this could explain why ulcers on smokers, in our study, tend to be colonized by *P. aeruginosa*, that wounds on smokers heal more slowly thereby giving the time to *P. aeruginosa* to settle in.

In the present study the 12-week outcome can be interpreted as a predictor for the long-term outcome. Kirsner et al[26] reported similar findings of a critical period of 3 months postgrafting, after which, if the graft was intact, it tended to remain so.

It is remarkable that even with aggressive treatment of *P. aeruginosa* the results evidently demonstrate a connection between *P. aeruginosa* and deterioration of skin grafts. We believe that the explanation lies in insufficient eradication of *P. aeruginosa* and that it is the persistence of *P. aeruginosa* that deteriorates the clinical outcome. From our clinic, Gjødsbøl et al[27], reported that more than half of the leg ulcers (52,2%) investigated in their study, concerning chronic venous leg ulcers, were colonized by *P. aeruginosa,* thus emphasizing the importance of this issue and the relevance in getting to the core of the problem. Also their results indicated that it was the same strain of *P. aeruginosa* that colonized the ulcers during an eight-week period, proposing that once the ulcer was colonized it remained so. To this should be mentioned that no antibiotics were administered in their study period.

The lack of successful antibiotic treatment is probably explained by ability of colonizing bacteria to establish themselves and proliferate in a biofilm. A biofilm is a multi cellular aggregate encased in a extracellular matrix of polysaccharides, protein, DNA etc, compared to single free swimming bacteria termed planktonic cells[28]. The clinical implications of bacterial biofilms are particularly pronounced in chronic infections[29]. In addition to being highly tolerant to antibiotics, biofilms are also impervious to the body's natural immune defence system[15]. *P. aeruginosa* and *S. aureus* are well recognised for forming chronic biofilm-based infections in their hosts. Normally radical debridement of the infected area is the treatment of choice in case of biofilm infections. In this study a thorough debridement was performed down to viable and visually non-infected tissue. Despite this, detection of *P. aeruginosa* prior to surgery, reduced graft take significantly. This indicates that *P. aeruginosa* resides deep down in the tissue, and is probably protected from antibiotics and the immune system due to biofilm formation. This is in accordance with a study by Fazli et al[30] showing a non-random distribution of *P. aeruginosa* and *S. aureus* where *P. aeruginosa* is found deeper into the tissue than *S. aureus.*

The sampling for microbiology of this study was performed by either a charcoal swab or by a biopsy. Several researchers have previously reported that surface swabs are considered equivalent to biopsy cultures[16] and that it reflects the microflora of deeper tissues[31]. However, this is up for debate. Bjarnsholt et al[18] indicated that bacteria are assembled in microcolony based structures found in bacterial biofilms and are far from evenly distributed within the wound, thereby implicating that cultures from a biopsy or swab are not likely to be representative for the total bacteriological load in the wound.

In the present study there was no correlation between the presence of haemolytic streptococci, isolated at least once from 12 weeks prior, to or during surgery, and the outcome of the STSGs. A part of the reason is, supposedly, due to our protocol that ensures rapidly administration of antibiotics in regards to haemolytic streptococci, in particular *S. pyogenes*. The reason why the antibiotic treatment seems to eradicate these strains rather than the *P. aeruginosa* probably lies in the fact that the haemolytic streptococci are in their planktonic state in order to spread into the tissue and therefore are susceptible to antibiotics.

The number of ulcers colonized by anaerobe bacteria in this study is probably not representative of the real burden of these, because of deficiencies in the bacteriologic investigations performed. The present study has some limitations due to the culturing methods. Conventional culturing often fails to identify all bacteria as some are hard to grow, some are fastidious or only present in certain parts of the wound. Previous studies have shown that *P. aeruginosa* is especially underestimated by culturing methods, probably due to their biofilm state[18], [20], [32], [33], but this accounts for other bacteria as well. We find that unidentified presence of *P aeruginosa* could explain some of the failures in the non-Pseudomonas group, but this remains to be pure speculation.

This study supports our hypothesis that *P. aeruginosa* in chronic venous leg ulcers, despite treatment, plays a considerable role in partial take or rejection of STSGs. Further studies on how to eliminate *P. aeruginosa* in this clinical situation and if this could improve the success rate will be needed. For biofilms, the lack of efficient antibiotic effect seems to depend on multiple mechanisms that can act together[28]. Further understanding of this might provide a rational basis for therapeutic manipulation of the wound environment that could ensure more successful skin grafting by eradicating *P. aeruginosa.* Given the limitations of this retrospective study we do recommend further studies to establish the role of bacteria in chronic wounds. In the light of this new information, we will strongly advise against transplanting chronic venous leg ulcers without a strategy how to eliminate *P. aeruginosa*.
